# Development of a tissue-specific bioscaffold for intestinal stem cell culture

**DOI:** 10.1371/journal.pone.0328898

**Published:** 2025-08-06

**Authors:** Sachin Kakar, Mathieu F. Derouet, Liyue Zhang, Connor J. Gillis, Frederikke Larsen, Arghya Paul, Lauren E. Flynn, Samuel Asfaha

**Affiliations:** 1 Department of Pathology and Laboratory Medicine, Schulich School of Medicine and Dentistry, The University of Western Ontario, London, Ontario, Canada; 2 Department of Medicine, Schulich School of Medicine and Dentistry, The University of Western Ontario, London, Ontario, Canada; 3 Verspeeten Family Cancer Centre, Cancer Research Laboratory Program, London, Ontario, Canada; 4 School of Biomedical Engineering, Faculty of Engineering, The University of Western Ontario, London, Ontario, Canada; 5 Department of Chemical and Biochemical Engineering, Faculty of Engineering, The University of Western Ontario, London, Ontario, Canada; 6 Department of Chemistry, Faculty of Science, The University of Western Ontario, London, Ontario, Canada; 7 Department of Anatomy and Cell Biology, Schulich School of Medicine & Dentistry, The University of Western Ontario, London, Ontario, Canada; 8 Department of Physiology and Pharmacology, Schulich School of Medicine & Dentistry, The University of Western Ontario, London, Ontario, Canada; Università degli Studi della Campania, ITALY

## Abstract

The generation of a tissue-specific intestinal hydrogel derived from the native intestine has the potential to support and promote the growth of intestinal organoids. In this study, we aimed to develop hydrogels derived exclusively from intestinal extracellular matrix (ECM) or composites comprised of intestinal ECM combined with alginate that allow for greater tuning of the hydrogel properties. A novel mouse intestinal decellularization protocol was developed and the ECM characterized. Our analyses demonstrate that our protocol effectively removed cellular and nuclear content while preserving key ECM components including collagens, glycosaminoglycans, fibronectin and laminin. When the decellularized small intestine (DSI) was used to generate hydrogels, the resulting ECM showed bioactivity as demonstrated by metabolic and pro-proliferative effects on NIH 3T3 murine fibroblasts. Importantly, our novel DSI hydrogels also supported murine intestinal and colonic organoid growth similar to Matrigel® controls. These studies demonstrate that murine tissue-specific DSI hydrogels can provide a supportive environment for the culture of intestinal and colonic organoids *in vitro*.

## Introduction

Over the last decade, the development of protocols for the establishment and growth of 3-dimensional (3D) multicellular organoids has revolutionized our ability to study epithelial biology in health and disease. Intestinal organoids are derived from tissues and established from either single intestinal stem cells (ISCs), or partially dissociated crypts [1]. Importantly, organoids recapitulate many anatomic, cellular, and functional features of the native small intestine [2]. For example, intestinal organoids contain an internal lumen that is surrounded by a polarized epithelial layer, and culture of intestinal cells as organoids allows for multicellular differentiation resembling the native tissue.

In order to successfully grow intestinal organoids, a 3D matrix that supports intestinal stem cells growth is required. Hydrogels (natural and/or synthetic) have previously been used as 3D matrices to culture adult stem cell types from different organs [3–5]. The properties of hydrogels can be carefully tuned by controlling the type of polymers used, the degree of crosslinking between polymer chains, and the incorporation of various biological cues [5–8]. Hydrogel composition and biomechanical properties can play important roles in modulating stem cell fate and cell differentiation [9–12].

A variety of hydrogels and hydrogel composites have previously been investigated for applications in intestinal organoid growth and tissue regeneration [1,9–13]. Most intestinal organoid protocols rely on Matrigel® as the extracellular scaffold, and currently this is considered the “gold standard” ECM for promoting organoid growth [2]. Matrigel® is a solubilized basement membrane extracted from Engelbreth-Holm-Swarm (EHS) mouse sarcoma cells, and is composed of collagen type IV, glycoproteins including laminin, entactin and nidogen, proteoglycans including heparan sulphate, and a variety of growth factors [14]. Although useful for establishing 3D cultures, Matrigel® also has several limitations. Specifically, the fact that it is tumour-derived and the lack of detailed knowledge of its constituents limit its use for clinical applications. Moreover, Matrigel® lacks tunability in its mechanical properties [15].

A cell-supportive extracellular matrix (ECM) is essential to allow intestinal epithelial cells to adhere, grow, and differentiate as multicellular organoids [16]. An alternative bioscaffold that is not tumor derived, and can provide structural support and biochemical cues that mimic the native ECM environment is therefore required to allow ISC proliferation and lineage-specific differentiation [16]. Biomaterials derived from tissue-specific ECM present a promising alternative for developing improved platforms for ISC culture and delivery [17]. Specifically, hydrogels incorporating decellularized small intestine (DSI) have the potential to enable cell encapsulation with high viability, analogous to Matrigel®, while also providing tissue-specific cues that help direct cell proliferation and differentiation [18,19].

In this study, our goal was to develop a new tissue-specific platform for the culture of ISC-derived organoids. First, we established and validated a new protocol for decellularizing mouse small intestine, while avoiding the use of ionic detergents that can deplete the ECM of growth factors and glycosaminoglycans (GAGs) [20,21], and can have cytotoxic effects even at very low concentrations [22–24]. Next, we established methods for generating hydrogels comprised exclusively of DSI by adapting pepsin-digestion protocols that have been reported in the literature for other tissue sources [18,25–30]. We subsequently performed *in vitro* studies to compare the growth of mouse intestinal organoids encaspulated in our new DSI hydrogels versus Matrigel® controls. Finally, to develop a platform that would enable greater tuning of the mechanical properties and avoid the need for enzymatic digestion to enable cell release, we investigated the cell-instructive effects of incorporating the DSI within alginate hydrogels as a new composite. Alginate has the capacity to reversibly crosslink under mild conditions and also support the viability of encapsulated cell populations in culture [31,32]. Thus, we report the successful derivation of new DSI hydrogels that can be used to culture murine small intestinal and colonic organoids.

## Materials and methods

### Decellularization of murine small intestine

All animal studies were approved by the Western University Animal Care Committee according to guidelines established by the Canadian Council on Animal Care. Mice were housed in the London Regional Cancer Program vivarium. Mice were exposed to a 12 h light-dark cycle, with water and regular chow *ad libitum*.

Adult male and female C57BL/6 mice (2–4 months in age) were euthanized by CO_2_ overdose, and the entire small intestine was surgically extracted by cutting at the pyloric sphincter and the ileocecal valve and transferred into Hank’s balanced salt solution (HBSS). The intestines from ~35–45 mice were pooled together to create large batches for tissue characterization and *in vitro* studies. The tissues were perfused with HBSS to remove the intestinal contents. Next, the intestines were cut longitudinally with a scalpel and scraped with a glass slide to remove any residual intestinal contents. Finally, the tissues were minced into ~2 mm^3^ pieces using surgical scissors.

For decellularization, all solutions were supplemented with 1% (v/v) antibiotic- antimycotic (ABAM) (Invitrogen, ON, Canada) and 0.27 mM phenylmethylsulfonyl fluoride (PMSF) (excluding the enzymatic digestion steps), and all incubation steps were performed in a 100 mL solution volume at 37°C under agitation on a Labnet 311DS orbital shaker control system (Labnet International, Inc., NJ, United States) at 120 rpm. The tissues were then subjected to three freeze-thaw cycles (from −80°C to 37°C) in 10 mM tris (hydroxymethyl)aminomethane (Tris) and 5 mM ethylenediaminetetraacetic acid (EDTA) in deionized water (dH_2_O) (pH 8.0). Solutions were replaced between each freeze/thaw cycle. After the third thaw, the samples were transferred into 50 mM Tris in dH_2_O supplemented with 1% (v/v) Triton X-100 (pH 8.0) for 24 hours. The samples were then rinsed three times for 20 minutes each in Sorenson’s phosphate buffer (SPB) rinsing solution composed of 0.55 M sodium phosphate dibasic heptahydrate (Na_2_HPO_4_•7H_2_O) and 0.17 M potassium phosphate (KH_2_PO_4_) in dH_2_O (pH 8.0). The samples were then enzymatically digested for 6 hours in SPB digest solution composed of 0.55 M Na_2_HPO_4_•7H_2_O, 0.17 M KH_2_PO_4_, and 0.049 M magnesium sulphate heptahydrate (MgSO_4_·7H_2_O) in dH_2_O (pH 7.3) supplemented with 300 U/mL deoxyribonuclease (DNase) Type II (from bovine pancreas) and 20 U/mL ribonuclease (RNase) Type III (from bovine pancreas). Next, the samples were incubated in 1% (v/v) Triton X-100 in 50 mM Tris buffer (pH 8.0) for 24 hours. Finally, the samples were rinsed three times for 20 minutes in SPB rinsing solution, followed by two rinses in dH_2_O for 30 minutes, and then frozen at −80°C and lyophilized using a Labconco Freezone 4.5 lyophilizer (Labconco, MO, United States) for 48 hours. The lyophilized DSI was cryo-milled into a fine powder by transferring the samples into a milling chamber with two 10 mm stainless steel milling balls. The chambers were submerged in liquid nitrogen for 3 minutes prior to milling for 3 minutes at 30 Hz (Retsch Mixer Mill MM 400 milling system). This cycle was repeated for a total of three times, and the samples were then stored at room temperature within a dessicator until further use.

### Characterization of murine DSI

#### Histology.

Native and decellularized small intestine samples (N = 3 cross-sections/batch, N = 3 independent decellularization batches) were embedded in Tissue-Tek OCT compound (Sakura Finetek, CA, United States) and snap frozen in liquid nitrogen in preparation for cryosectioning (7 μm sections) with a Leica CM3050 S cryostat (Leica Microsystems Inc., ON, Canada). Sections were stained with 4’,6-diamidino-2-phenylindole (DAPI) in fluoroshield mounting medium (Ab104139, Abcam) or hematoxylin and eosin (H&E) to visualize cell nuclei, picrosirius red to visualize collagen, or toluidine blue to visualize glycosaminoglycans (GAGs), following standard protocols. DAPI images were obtained using an EVOS FL fluorescence microscope (Thermo Fisher Scientific Inc., ON, Canada). Toluidine blue staining was visualized using an EVOS XL Core microscope (Thermo Fisher Scientific Inc., ON, Canada), and the picrosirius red stained samples were imaged using a Nikon Optiphot polarizing microscope (Nikon Instruments Inc., NY, United States).

#### PicoGreen assay.

The PicoGreen® assay was used to assess the efficacy of decellularization by comparing the double stranded DNA (dsDNA) content in the fully processed DSI samples to native tissue controls. DNA was extracted from the samples using the DNeasy Blood & Tissue Kit (Qiagen, Germany), following the manufacturer’s protocols. dsDNA content within the native and decellularized tissue samples was quantified using the Quant-iT PicoGreen® assay (Molecular Probes, Ontario).

#### Biochemical assays.

The hydroxyproline and dimethylmethylene blue (DMMB) assays were used to quantify the hydroxyproline content as a measure of total collagen content and the sulphated GAG (sGAG) content within the decellularized and native tissue samples, respectively. The assays were performed following published protocols [33] with the absorbance read using a CLARIOstar® microplate reader. Values were normalized to the dry weight of each sample.

#### Immunofluorescence staining.

Native and decellularized mouse intestine samples (n = 3 cross-sections/batch, N = 3 independent decellularization batches) were embedded in Tissue-Tek OCT compound (Sakura Finetek, CA, United States) and cryosectioned, as described above. The sections were fixed in acetone for 10 minutes at −20°C and blocked in 10% goat serum in tris-buffered saline with 0.1% tween (TBST) for 1 hour at room temperature. The sections were then incubated overnight at 4°C with primary antibodies against collagen type I (dilution 1:100 in TBST with 2% BSA, Ab34710, Abcam, ON, Canada), collagen type IV (dilution 1:100, ab6586, Abcam), fibronectin (dilution 1:150, ab23750, Abcam), and laminin (dilution 1:200, ab11575, Abcam). Next, an anti-rabbit secondary antibody conjugated to Alexa Fluor 594 (dilution 1:200, ab150080, Abcam) was added and the samples were incubated for 1 hour at room temperature. Samples were then mounted in fluoroshield mounting media with DAPI. Images were acquired with an EVOS FL fluorescence microscope (Thermo Fisher Scientific Inc., ON, Canada).

### Establishment of hydrogels from murine intestinal ECM

To generate the DSI pre-gel solution, the cryomilled DSI was added at a concentration of 25 mg/mL (based on dry mass) to a sterile solution of 1 mg/mL porcine pepsin (3200–4500 mU/mg protein) in 0.05 M hydrochloric acid to obtain a total volume of 5 mL. The samples were digested for 24 hours at room temperature under agitation at 100 rpm. Following digestion, while on ice, 1:10 volume of 10X PBS was added and the solutions were neutralized with 1 M sodium hydroxide. The resultant solutions were stored at 4 °C and kept on ice during use.

To generate the hydrogels comprised exclusively of intestinal ECM, the 22.73 mg/mL pepsin-digested DSI solution was combined in a 1:1 ratio with sterile filtered H_2_O to obtain a final ECM concentration of 11.3 mg/mL. The diluted samples were pipetted in 50 μL droplets into a 24-well plate and crosslinked through incubation at 37 °C for 1 hour, to allow self-assembly of the collagen within the samples. Matrigel® gels were similarly fabricated as a control, following the manufacturer’s protocols.

### Rheological testing

Rheological testing was performed to assess the storage (G’) and loss modulus (G”) of the DSI hydrogels in comparison to Matrigel® using a HAAKE Modular Advanced Rheometer System (MARS) equipped with a P20/Ti titanium plate. Hydrogels were prepared as previously described. The neutralized pre-gel was then transferred to a syringe mould and incubated for 1 h at 37°C to form the hydrogel. The hydrogels were then gently extruded from the syringe mold onto the testing platform. Testing was performed at 37°C to simulate biological conditions using a shear stress amplitude sweep of 0.1 to 1 x 10^4^ Pa.

### Generation of composite alginate-based hydrogels

Composite alginate + DSI hydrogels were generated by combining the pepsin-digested DSI with alginate, to enable ionic crosslinking under mild conditions using a calcium chloride solution. Importantly, this crosslinking process can easily be reversed by chelating the cations using citrate or EDTA, to release the cells without the need for enzymatic digestion [34]. Control hydrogels were fabricated from alginate alone. Alginate was prepared by dissolving alginic acid sodium salt, low viscosity (Alfa Aesar, B25266) in sterile filtered H_2_O to obtain a 2% alginate concentration (w/v). The alginate solution was decontaminated for cell culture by heating it to 98°C for 30 minutes and cooled at room temperature before use. Composite alginate-ECM gels were fabricated by combining 2% (w/v) alginate with pepsin-digested DSI or sterile filtered H_2_O in a 1:1 ratio. The samples were pipetted in 50 μL droplets into a 24-well plate and immersed in 2% (v/w) calcium chloride for 1 hour at 37°C to crosslink the alginate and then washed with PBS to remove excess calcium chloride.

### Cell culture and encapsulation

A simplified cell culture model was first used to validate that the pepsin-digested DSI generated with the novel decellularization protocol would have bioactive effects on cell populations encapsulated within the hydrogels, prior to moving on to the more complex and heterogeneous cell populations within the organoid cultures. More specifically, the viability, spreading and metabolic activity of NIH 3T3 cells encapsulated in the DSI hydrogels were assessed in comparison to cells encapsulated in Matrigel® as a control. In addition, similar *in vitro* studies were performed to compare NIH 3T3 cells encapsulated in the alginate + DSI composites to alginate alone controls.

NIH 3T3 cells at passage 3–7 were encapsulated within 50 μL hydrogels prepared in 12-well cell culture inserts (Greiner Bio-one, Germany), following the methods described above, at a density of 1.0 x 10^6^ cells/mL. Following gelation, the samples were cultured in proliferation medium composed of DMEM (Wisent bioproducts, CAT# 319–005-CL) supplemented with 10% fetal bovine serum (Gibco®, Invitrogen, ON, Canada) and 1% penicillin/streptomycin (Gibco®, Invitrogen, ON, Canada) in a 5% CO_2_ humidified incubator at 37°C, with media changes every 2–3 days.

### Confocal analysis

The viability of the encapsulated NIH 3T3 cells was assessed using the LIVE/DEAD® Viability/Cytotoxicity Assay (Invitrogen CAT#L3224) with analysis by confocal microscopy at 24 hours, 3 days, and 7 days post-encapsulation. Live cells were identified through Calcein AM staining (green) and dead cells were labeled using ethidium homodimer-1 (EthD-1) (red). Non-overlapping images were taken using a 5X objective across the entire cross-section of each gel at defined depths ranging from 70 μm to 170 μm using a Zeiss LSM800 Confocal Microscope.

### Metabolic activity analysis

The metabolic activity of the encapsulated 3T3 cells was assessed at 24 hours, 3 days, and 7 days post-encapsulation using the CyQUANT™ MTT Cell Viability Assay kit (Thermo Fisher Scientific Inc. CAT#V13154) (N = 3 replicate hydrogels/group, N = 5 experimental repeats for Day 1 to Day 7 studies, N = 3 for Day 7 only studies), following previously published methods [35].

### Intestinal and colonic organoid culture

Adult male and female C57BL/6 mice (2–4 months in age) were euthanized by CO_2_ overdose and their colon and intestines were surgically extracted. The methods for organoid culture were adapted from methods previously described [36]. Briefly, the intestinal and colonic tissues were cut into 0.5 mm pieces and washed with PBS. Following PBS washing, the intestinal and colonic tissues were resuspended in 10 mL of 2.5 mM EDTA in PBS and incubated at 4 °C on a rotator for 45 minutes.

After centrifugation, the crypts were counted, and then resuspended in 50 μL droplets of Matrigel® (Fisher CAT#356231). After polymerization, 500 μL of Advanced DMEM/F12 supplemented with penicillin/streptomycin, 10 mM HEPES, 1x Glutamax, 1x B27, 1x N2, and 1 mM of N-acetylcysteine, 50 ng/mL murine recombinant EGF (Thermo Fisher Scientific), 100 ng/mL Noggin (PeproTech), and 1 ug/mL R-spondin1 purified from HA-R-Spondin1-Fc 293T cells (Trevigen) was added to each well. For the colonic cultures, 50% Wnt3a conditioned medium was added to the medium as previously described [37]. Organoids were grown at 37°C in a humidified atmosphere containing 5% CO_2_. each well. Media was changed every 2 days.

### Encapsulation of intestinal and colonic organoids in DSI-based hydrogels

Intestinal organoids were first cultured and maintained in Matrigel® for 14 days before passaging into the hydrogels. To release the organoids, the Matrigel® was physically disrupted through gentle pipetting and the samples were centrifuged for 5 minutes at 250xg. The organoid cultures were then re-seeded in either the pepsin-digested DSI hydrogels or Matrigel®, or the alginate-based hydrogels (alginate + DSI, alginate alone), following the methods described above.

To assess the viability of intestinal stem cells in the DSI hydrogels, the organoids grown for 14 days in DSI hydrogels were then passaged back into fresh DSI hydrogels or Matrigel®. Organoid area was measured using the ImageJ software. For organoid area quantification, the area of n > 100 organoids per group was quantified/timepoint using positive pixel counting and used to estimate the average fold-change in size from days 1–7 as a measure of organoid growth.

### Histology and Alcian blue staining for intestinal organoids

Intestinal and colonic organoids cultured for 7 days in either Matrigel® or DSI hydrogels were fixed for 1 h at room temperature in 4% formalin, prior to embedding in 3% (v/w) agarose. The agarose gels containing the encapsulated organoids were embedded in paraffin and sectioned (5 μM). Tissue sections were deparaffinized in xylene and rehydrated in decreasing concentrations of ethanol. For histology, tissues were stained with CAT hematoxylin (Biocare Medical) and Eosin Y (Sigma) and were subsequently rehydrated and mounted using Permount (Fisher Scientific) for imaging.

For Alcian blue staining, sections were deparaffinized, hydrated in distilled water, incubated in 3% glacial acetic acid solution for 3 minutes, followed by a 30-minute incubation in Alcian-blue stain (1% Alcian blue in 3% glacial acetic acid; pH 2.5). Sections were counterstained with Nuclear Fast Red (0.1%; Sigma, USA) for 5 min, rinsed in water, dehydrated, and mounted for visualization.

### Quantitative RT-PCR

Total RNA was extracted from the intestinal organoids cultured for 7 days in the DSI hydrogels or Matrigel® using a Qiagen RNeasy Kit (Qiagen). RNA concentration was determined using a NanoDrop UV-Vis Spectrophotometer (Thermo Fisher Scientific). cDNA was synthesized using 1 µg of RNA and was performed using the iScript cDNA Synthesis Kit (Bio-Rad). RT-qPCR was carried out in triplicate using PowerUp SYBR Green Master Mix (Thermo Fisher) and ViiA QuantStudio 5 (Thermo Fisher). Gene expression levels were quantified using the ddCT method and normalized to the Ct value of GAPDH. See S1 Table for the complete list of primers used.

### Statistical analyses

All statistical analyses were carried out by t-test or two-way ANOVA as detailed in the figure captions using GraphPad Prism 7.0. All numerical data are expressed as mean ± standard deviation (S.D.). Differences with p < 0.05 were considered statistically significant.

## Results

### Characterization of decellularized intestinal tissues

Our first goal was to establish a mouse intestinal decellularization protocol that removes cellular content while preserving the ECM composition. The small intestines from multiple adult mice were pooled and processed together to generate large batches of decellularized tissue that could be used for hydrogel fabrication. Briefly, our new 4-day decellularization protocol involved freeze-thaw cycles in a hypotonic buffer to promote cell lysis, extraction with the non-ionic detergent Triton X-100, and enzymatic digestion with DNase and RNase to deplete residual nucleic acids.

**Fig 1:**
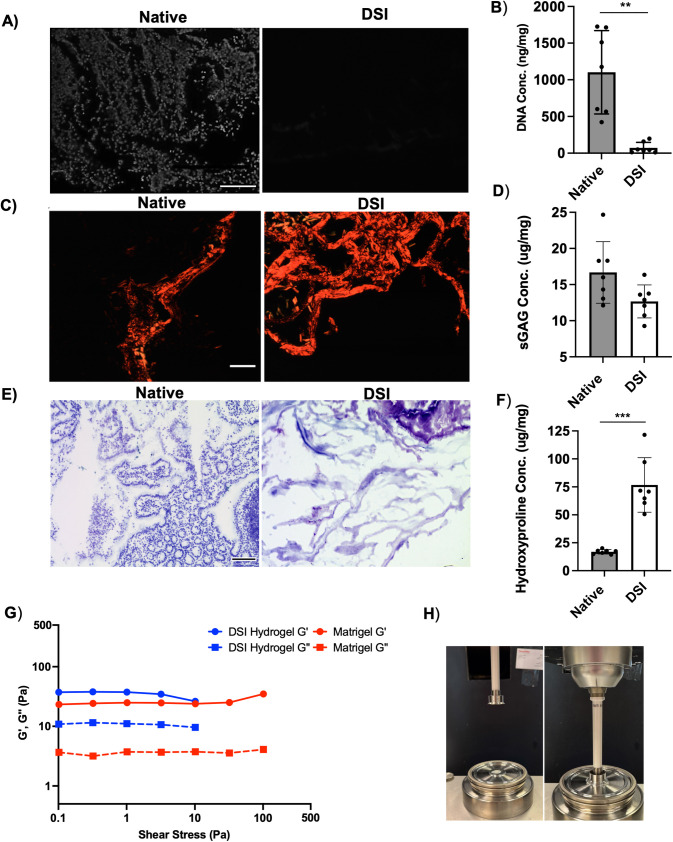
Decellularization protocol effectively removed cellular contents from the mouse small intestine. A) Representative DAPI nuclear staining (shown in grayscale) of native mouse small intestine (left panel) versus DSI (right panel) (n = 3 cross-sections/decellularization batch N = 3 independent decellularization batches). Scale bar = 100 μm. B) Quantitative analysis of double-stranded DNA (dsDNA) content using the PicoGreen assay. Values are reported based on dry weight. (N = 7 independent decellularization batches). Mean ± S.D, Paired *t*-test; **p < 0.01. C) Representative polarized light microscopy images of picrosirius red staining showing that the DSI contained a qualitatively denser network of collagen fibers than the native tissue samples. (n = 3 cross-sections/decellularization batch, N = 3 independent decellularization batches). Scale bar = 500 μm. D) Quantification of total collagen content by the hydroxyproline assay. Values are reported based on dry weight. (N = 7 independent decellularization batches). Mean ± S.D, Paired *t*-*t*est; ***p < 0.001. E) Representative toluidine blue staining showing GAGs (purple) and nucleic acids (blue) in the native tissue samples versus DSI. (n = 3 cross-sections/decellularization batch, N = 3 independent decellularization batches). Scale bar = 100 μm. F) Quantitative analysis of sGAG content with the DMMB assay. Values are reported based on dry weight. (N = 7 independent decellularization batches). Mean ± S.D, Paired *t*-tes*t*. G) Representative rheological testing depicting the storage (G’) and loss (G”) moduli of the DSI hydrogels and Matrigel® controls (n = 5-6). Inset shows images taken during rheological testing. DSI hydrogels have a slightly greater storage and loss modulus when compared to Matrigel®, indicating they are more elastic and resistant to deformation at lower shear stresses. However, the DSI hydrogels were less resistant to higher shear stresses, degrading faster than Matrigel®. H) Pictures of the rheometer during rheological testing.

To confirm the effectiveness of cell extraction, DAPI staining was performed to visualize cell nuclei within the DSI samples and compared to native tissue controls (Fig 1A). Notably, no visible nuclei were detectable in the tissues following decellularization. Representative H&E staining images of the native small intestine versus DSI further confirmed that the new protocol effectively removed cellular components (S1 Fig, right panel), to yield a collagen-rich ECM. To further validate these findings, the PicoGreen assay was used to quantify the dsDNA content in the native small intestine and DSI samples (Fig 1B). A significant reduction in the dsDNA content was observed following decellularization, with an average decrease of 92 ± 10% relative to native tissue controls.

Following decellularization, the collagen content of the intestinal tissue samples was assessed. The samples were stained using picrosirius red and imaged using polarized light microscopy to visualize the network of collagen fibers. In general, a denser network of collagen fibers stained red was visualized in the DSI tissues relative to the native tissue controls (Fig 1C). In addition, a hydroxyproline assay was used to quantify the collagen content in the native tissue versus DSI samples (Fig 1D). We observed a significant increase in the relative collagen content following decellularization, consistent with the removal of cells and other ECM constituents. Qualitative assessment of GAGs via toluidine blue staining revealed similar levels of purple staining in the DSI samples versus the native small intestinal tissue (Fig 1E). Using a DMMB assay, we confirmed that there was no significant difference in the sGAG content in the DSI versus native small intestine samples (Fig 1F). Similarly, immunofluorescence staining of ECM components confirmed that collagen I, collagen IV, fibronectin, and laminin were retained following decellularization (S2 Fig).

Lastly, we performed rheological testing to assess the mechanical stability and rheological properties of the DSI hydrogels relative to Matrigel® controls (Fig 1G). Both formulations were found to form stable hydrogels, as a tan(δ) value of less than one indicates more solid-like behavior [38]. At a shear stress of 1 Pa, the representative DSI hydrogel was found to have a tan(delta) value of 0.20, while the representative Matrigel® sample had a value of 0.15. Additionally, rheological testing showed that the DSI hydrogels had a slightly greater storage and loss modulus than Matrigel®, meaning that the DSI hydrogels were more elastic and resistant to deformation at lower shear stresses. However, the Matrigel® controls were found to be more resistant to greater shear stresses. This may be beneficial from a cell culture perspective, as the DSI hydrogels could be easily broken up to remove encapsulated cells. Additionally, overall, the DSI hydrogels were found to be more consistent in their rheological properties compared to Matrigel® (S1B Fig).

### In vitro assessment of intestinal ECM hydrogel bioactivity using encapsulated NIH 3T3 cells

**Fig 2:**
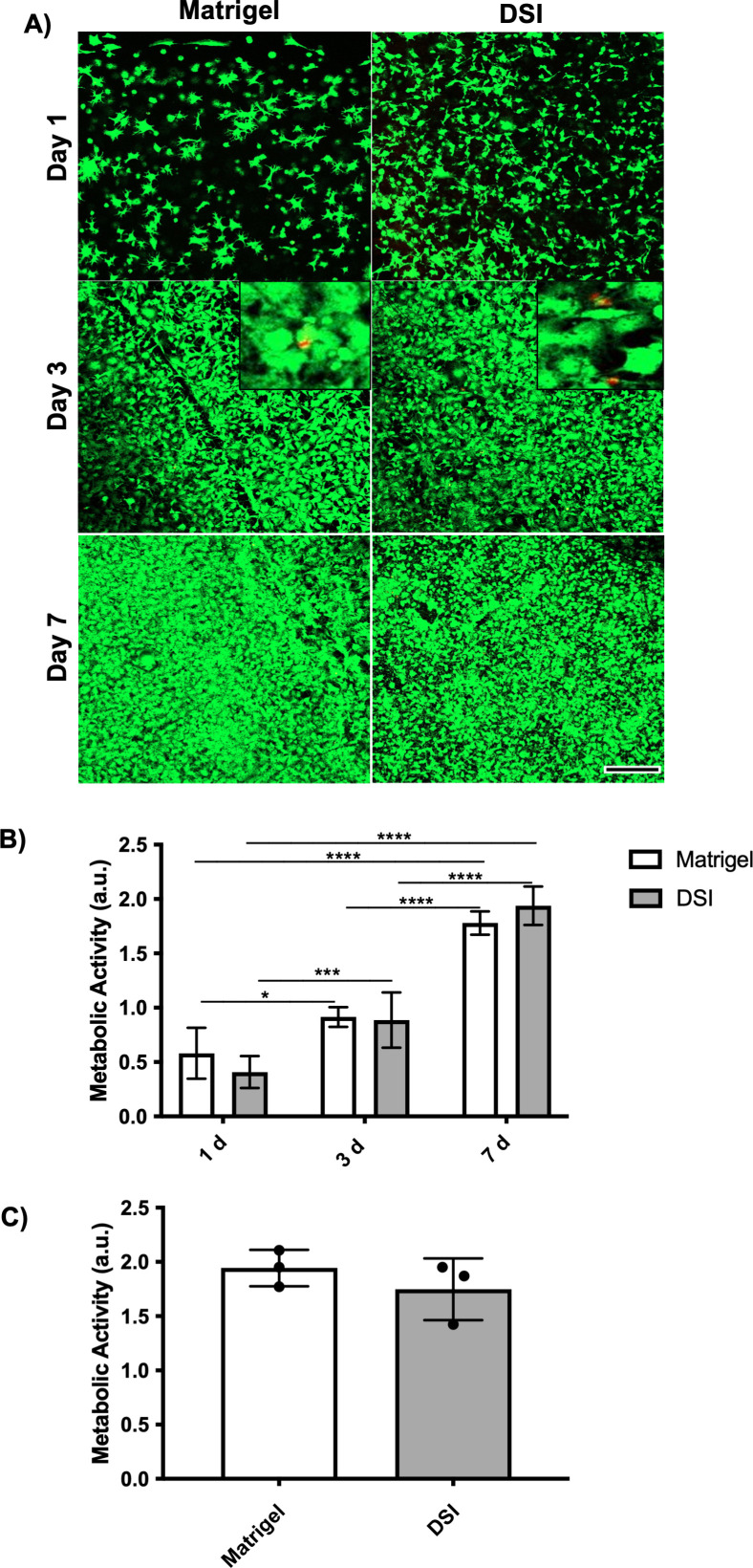
DSI hydrogel and Matrigel® promote similar growth and viability of murine fibroblasts. A) Representative confocal microscopy images showing stained calcein^+^ live (green) and EthD-1^+^ dead (red) 3T3 fibroblasts within DSI hydrogels or Matrigel® controls. High cell viability was maintained with both platforms over the 7-day culture period. Cell spreading was observed at all time points in both groups. Insets on Day 3 show the presence of infrequent red (dead) cells. (n = 3 hydrogels per timepoint/trial, N = 3 trials with independent ECM batches). Scale bar = 500 μm. B) Quantification of metabolic activity using a MTT assay showed similar metabolic activity in 3T3 cells encapsulated within the DSI hydrogels or Matrigel® across all time points. Higher metabolic activity levels were observed at day 7 as compared to day 1 and 3 for both groups, consistent with cell proliferation. (N = 5 separate 3T3 encapsulations). Mean ± S.D, Two-way ANOVA; *p < 0.05, ***p < 0.001, ****p < 0.0001. C) Metabolic activity of 3T3 cells encapsulated in DSI hydrogels prepared from different ECM batches, showing consistency in the response to the developed bioscaffolds and comparable metabolic activity levels to Matrigel® controls at 7 days post-encapsulation. (N = 3 independent decellularized and pepsin-digested DSI batches). Mean ± S.D, Unpaired *t*-test. Me*t*abolic activity was measured by absorbance values in arbitrary units (a.u.) (B-C).

To confirm the bioactivity of the ECM derived using our novel decellularization protocol, a LIVE/DEAD assay with confocal imaging was performed to assess the viability and spreading of NIH 3T3 murine fibroblasts encapsulated within the DSI hydrogels compared to Matrigel® controls at days 1, 3, and 7 post-encapsulation (Fig 2A). Qualitative assessment revealed that both groups showed similar high viability and cell spreading, indicative of cell attachment to ECM components over the 7-day culture period. The cell density qualitatively appeared to increase over time in both groups.

To quantitively compare cell viability between the groups, an MTT assay was performed to measure the metabolic activity of the encapsulated 3T3 cells (Fig 2B). Consistent with the imaging results, there were no significant differences in the metabolic activity between the groups at any of the time points examined. Furthermore, there was a significant increase in metabolic activity on day 7 compared to days 1 and 3 for both groups, consistent with cell growth.

To assess potential batch-to-batch variability in the decellularization process, NIH 3T3 cells were encapsulated in DSI hydrogels prepared with ECM from 3 independent decellularization batches, or in Matrigel® as a control, and cultured for 7 days (Fig 2C). Importantly, MTT analysis of metabolic activity at day 7 showed consistent results across all 3 batches, with no significant difference relative to the Matrigel® controls.

### In vitro assessment of murine intestinal and colonic organoids cultured in DSI hydrogels and Matrigel®

**Fig 3:**
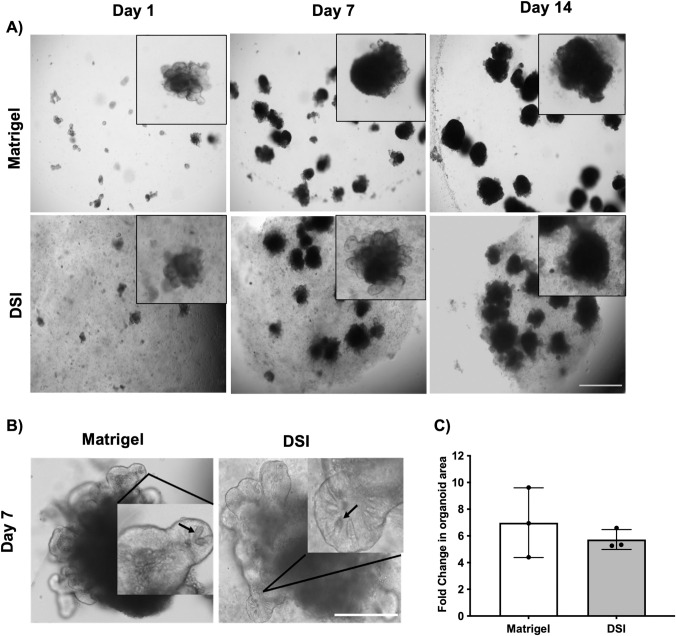
DSI hydrogels promote the growth of mouse intestinal organoids *in vitro.* A) Time course of mouse intestinal organoid growth is shown. Insets show high power images of single organoids over the culture period. (N = 3 independent ECM batches). Scale bar = 1 mm. B) Representative brightfield microscopy images of organoids grown in Matrigel® versus DSI hydrogels at day 7. Images of organoids encapsulated within both Matrigel® and DSI hydrogels revealed granule-containing cells in the budding crypts, consistent with the presence of Paneth cells (black arrows). Scale bar = 200 μm. C) Quantification of organoid size in Matrigel® versus DSI hydrogels from days 1 to 7, showing similar organoid growth in both groups. (n ≈ 175 organoids per group were quantified/timepoint, N = 3 independent organoid cultures). Mean ± S.D, Unpaired *t*-test.

To assess the ability of the DSI hydrogels to promote intestinal organoid growth, primary mouse intestinal organoids were first cultured in Matrigel® prior to being passaged and cultured within Matrigel® or DSI hydrogels (Fig 3). Intestinal organoids were cultured for 14 days and imaged on days 1, 7, and 14 post-encapsulation (Fig 3A). Qualitatively, organoid growth was similar between the DSI hydrogels and Matrigel® cultures over 14 days. Microscopy imaging on day 7 post-encapsulation revealed budding of organoids grown in either Matrigel® or the DSI hydrogels (Fig 3B). Organoid area was calculated using ImageJ on day 1 and 7 post-encapsulation. The fold change in organoid area on day 7 relative to day 1 was similar in the DSI hydrogels and Matrigel® controls (Fig 3C).

**Fig 4:**
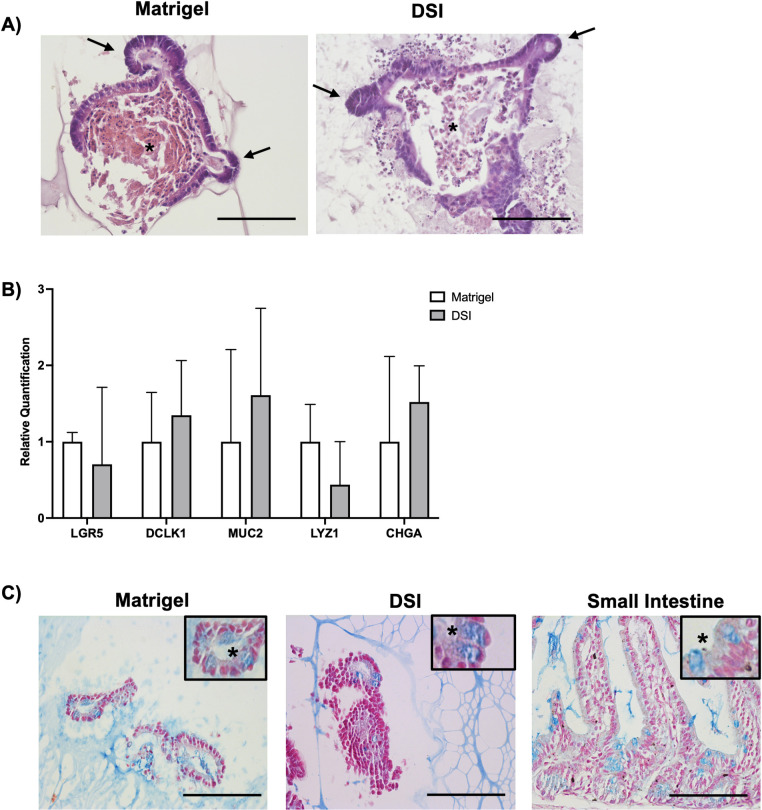
DSI hydrogels show a similar capacity to Matrigel® to support intestinal organoid cultures, including cell differentiation. A) Representative H&E staining of organoids grown in Matrigel® versus DSI hydrogels at day 7 post-encapsulation. Arrows denote intestinal budding that mimics crypts within the native intestine. Dead epithelial cells (*) sloughed off into the lumen of the organoids are seen in the center (n = 3 cross-sections, N = 3 independent organoid cultures). Scale bar 50 = μm.**B)** Relative mRNA expression of Lgr5 (stem cell), Dclk1 (tuft cell), Muc2 (goblet cell), Lysozyme (Paneth cell), and Chromogranin A (neuroendocrine cell) in the organoids cultured in Matrigel® (n = 3) or the DSI hydrogels, showing similar differentiation between the two platforms (n = 3). Data are presented as mean ± SD Statistical significance was assessed by Student’s unpaired t-test. C) Representative images of Alcian blue staining of sections from mouse small intestine, as well as organoids cultured in Matrigel® and DSI hydrogels. The presence of goblet cells is highlighted by the blue vesicles (see asterisk). Scale bars = 100 µm.

Representative H&E staining of organoids grown in Matrigel® and DSI at day 7 revealed a multicellular budding epithelial layer (Fig 4A). Building from this, we performed RT-qPCR to compare the gene expression levels of the markers Lgr5 (stem cell), Dclk1 (tuft cell), Muc2 (goblet cell), Lysozyme (Paneth cell), and Chromogranin A (neuroendocrine cell) in the organoids cultured in Matrigel® and the DSI hydrogels (Fig 4B). We observed no significant differences in the expression levels of any of the genes when comparing the Matrigel® and DSI groups, suggesting that the differentiation response was similar between the two platforms. To corroborate our gene expression data, we performed Alcian blue staining to identify goblet cells in both our Matrigel® and DSI gels, which also showed qualitatively similar differentiation in both groups, observed in all cultures at day 7 (Fig 4C).

**Fig 5:**
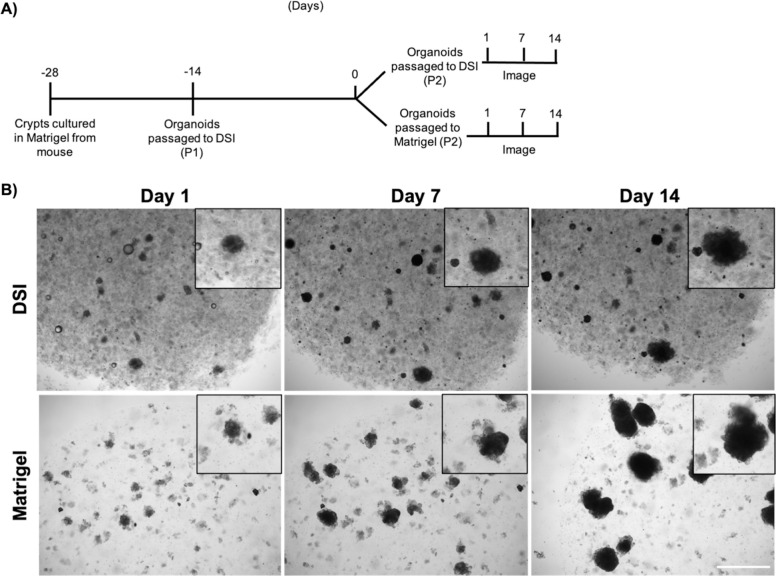
Organoids remain viable following passaging from the DSI hydrogels. A) Schematic of the timeline for organoid culture and passaging. B) Representative brightfield microscopy images of mouse intestinal organoids cultured in the DSI hydrogels and passaged into new DSI hydrogels or Matrigel®. Images were taken at days 1, 7, and 14 post-passaging. Intestinal organoids passaged into either DSI hydrogels or Matrigel® were followed over 14 days, with fewer organoids growing in the DSI hydrogel group versus the Matrigel®. (N = 3 independent ECM batches). Scale bar = 1 mm.

**Fig 6:**
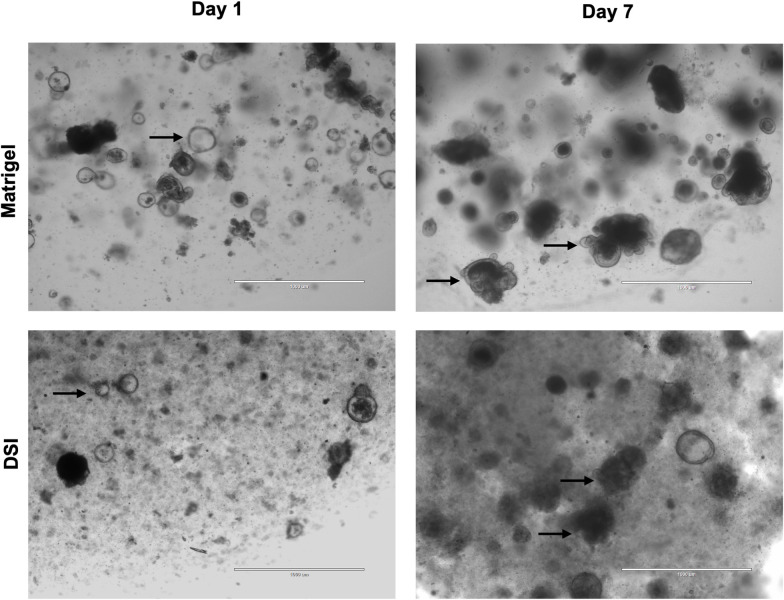
DSI hydrogels promote the growth of mouse colonic organoids *in vitro.* Representative brightfield microscopy images of organoids grown in Matrigel® versus DSI hydrogels at day 1 and day 7. At day 1, the organoids in both cultures displayed a cystic morphology (black arrows), while some budding was detected (black arrows) at day 7 in both the Matrigel® and DSI groups. Scale bar = 1 mm.

Next, mouse intestinal organoids grown in the DSI hydrogels were passaged into either Matrigel® or DSI hydrogels and imaged on days 1, 7, and 14 to assess growth and viability (Fig 5A). The organoids encapsulated within the DSI hydrogels and Matrigel® both showed growth over the 14-day culture period. Notably, we observed that fewer organoids increased in size in the DSI hydrogels when compared to the organoids grown in Matrigel® (Fig 5B). Finally, we tested if murine colonic organoids could grow in the DSI hydrogels. Analogous to the intestinal organoids, we cultured colonic organoids in Matrigel® and then passaged them into Matrigel® and DSI gels. After 7 days of culture, we observed organoid growth and budding in both Matrigel® and the DSI hydrogels (Fig 6).

### The proliferation of NIH3T3 cells, but not intestinal organoids, was enhanced in the compsite alginate + DSI hydrogels

**Fig 7:**
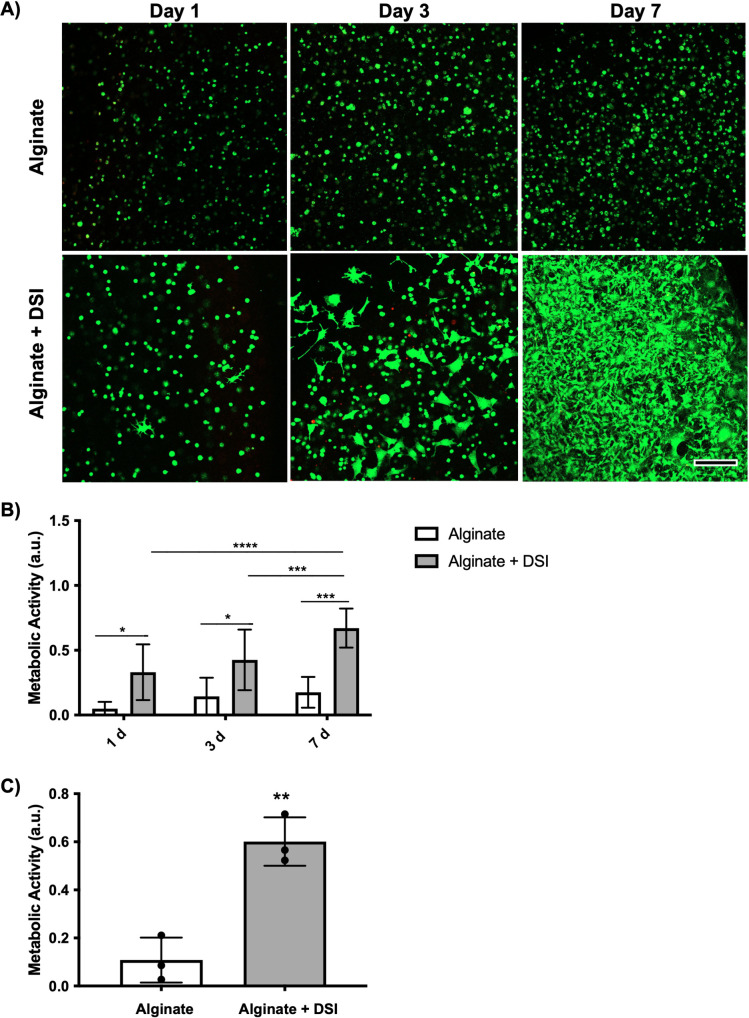
Inclusion of pepsin-digested DSI within alginate hydrogels promotes cell spreading and growth of encapsulated NIH 3T3 murine fibroblasts. A) Representative confocal images showing calcein^+^ live (green) and EthD-1^+^ dead (red) 3T3 fibroblasts in all hydrogels. High cell viability was maintained throughout the culture period. Cell spreading was observed at all time points in the alginate + DSI hydrogels, but not in the alginate alone control group. (n = 3 hydrogels per timepoint/trial, N = 3 trials with independent ECM batches). Scale bar = 500 μm. B) Quantification of metabolic activity using a MTT assay revealed higher metabolic activity levels in the alginate + DSI group relative to the alginate alone at all time points (N = 5 separate 3T3 encapsulations). Mean ± S.D, Two-way ANOVA; *p < 0.05, **p < 0.01, ***p < 0.001, ****p < 0.0001. C) Quantification of metabolic activity through the MTT assay showed consistently higher levels of metabolic activity at 7 d in the alginate + DSI samples relative to the alginate alone across three separate ECM batches. (N = 3 independent decellularized and pepsin digested ECM batches). Mean ± S.D, Unpaired *t*-test. Metabolic ac*t*ivity was measured by absorbance values in arbitrary units (a.u.).

To develop an alternative cell encapsulation platform that retains the bioactivity of DSI and allows for more tunable mechanical properties, we incorporated the pepsin-digested DSI within alginate to form a composite hydrogel product. Two groups of hydrogels were then compared: 1% alginate and 1% alginate + DSI, with crosslinking performed using calcium chloride. Analogous to our studies above using the hydrogels comprised exclusively of DSI, we once again used encapsulated 3T3 murine fibroblasts to confirm that the DSI within the composites showed similar bioactivity. LIVE/DEAD imaging analyses confirmed that all groups had high viability across the 7-day culture period (Fig 7A). Cell spreading was observed in the alginate + DSI group, whereas the alginate alone group showed a spherical cell morphology, suggesting the incorporated ECM provided cell-adhesive cues. Using the MTT assay, we observed that the fibroblasts encapsulated within the alginate + DSI composites showed higher metabolic activity when compared to cells encapsulated in the alginate alone at days 1, 3, and 7 post-encapsulation (Fig 7B). The metabolic activity of cells encapsulated within the alginate + DSI hydrogels was higher at day 7 versus days 1 and 3, consistent with cell growth in the composites but not in the alginate alone. Similar results were observed with composites generated from three different DSI batches (Fig 7C).

**Fig 8:**
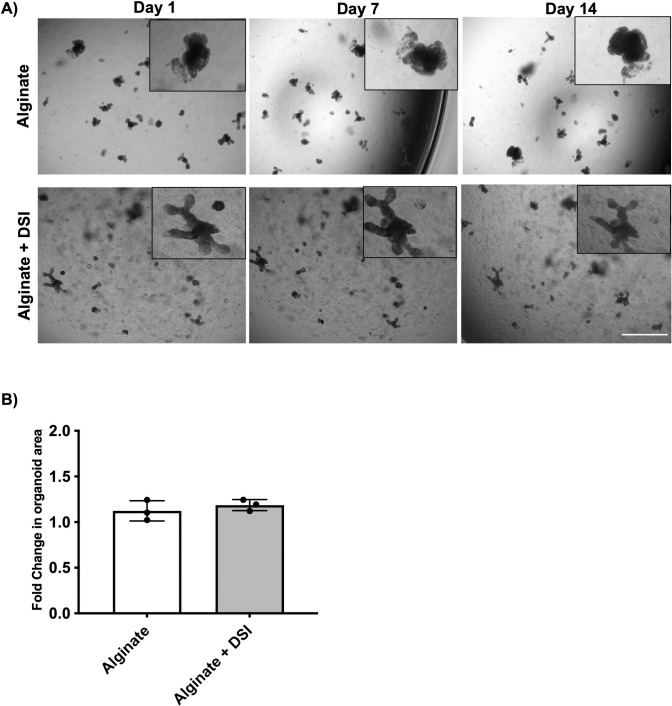
Incorporation of DSI within alginate hydrogels is insufficient to promote growth of mouse intestinal organoids *in vitro.* A) Time course of mouse intestinal organoids showed a lack of organoid growth over 14 days in both the alginate + DSI group and alginate alone group. (N = 3 independent ECM batches). Scale bar = 1 mm. B) Analysis of the change in size of organoids cultured in alginate versus alginate + DSI from days 1 to 7 post-encapsulation. The analysis showed no organoid growth in both hydrogels. (n ≈ 100 organoids per group/timepoint, N = 3 independent organoid cultures). Mean ± S.D, Unpaired *t*-test.

To assess whether the alginate-based hydrogels could support the growth of intestinal organoids, primary mouse intestinal organoids were cultured in Matrigel® prior to being passaged and encapsulated within alginate alone or composite alginate + DSI hydrogels. Intestinal organoids were imaged using brightfield microscopy on days 1, 7, and 14 post-encapsulation (Fig 8A). In general, the organoids appeared morphologically similar within the alginate + DSI hydrogels versus alginate alone hydrogels. Specifically, we detected no observable change in the size or structure of the organoids over time. Organoid area calculated using ImageJ on days 1 and 7 post-encapsulation confirmed the fold change in organoid area was similar in the alginate + DSI hydrogels and alginate alone group, with neither group showing a significant increase in size over time (Fig 8B).

## Discussion

Development of a well-defined bioscaffold that is not cancer cell derived and suitable for clinical application of organoids remains an unmet need [25]. In this study, we describe a novel protocol for derivation of hydrogel platforms from murine DSI. The native intestinal crypt is comprised of an ECM that includes numerous bioactive proteins including laminin, collagen, proteoglycans, and fibronectin [16]. This complex network of proteins and polysaccharides forms the supporting structure for the intestinal epithelium while also providing essential biochemical cues for proliferation and differentiation [16]. Thus, biomaterials derived from tissue-specific ECM provide a promising alternative for ISC culture and *in vivo* delivery due to their innate bioactivity and ability to be degraded through cell-mediated mechanisms [39]. Previously, Giobbe et al. described successful growth of endoderm-derived human and mouse organoids using gels derived from decellularized porcine small intestine mucosa/submucosa [18]. To our knowledge, however, this is the first report of the development of hydrogels derived from murine intestinal ECM used to successfully grow and maintain murine intestinal organoids.

In order to develop tissue-specific bioscaffolds for various applications, decellularization is often used to isolate the ECM from native tissues [18,25,40,41]. Decellularization removes cellular components that can induce an immunogenic response while preserving the structure and composition of the native ECM [42]. The extraction of cells, however, requires methods that are tailored to the physical and biochemical properties of each unique tissue [43], and their effectiveness depends on factors such as the tissue cellularity, density, and lipid content [20]. In contrast to previous reports [18,20,22,44–47], we developed a novel decellularization protocol that avoids the use of stronger ionic detergents that often cause greater loss of soluble ECM components such as growth factors and GAGs [20,21], and are potentially cytotoxic [22–24]. We found that our protocol was highly effective at extracting nucleic acids from the tissues and comparable to other protocols in the literature that used the ionic detergent SDS to decellularize mouse small intestine [44].

Notably, GAGs can sequester growth factors, play a key role in maintaining tissue hydration [16,48] and are known to modulate cellular processes in the intestinal crypt through direct interaction with cell surface receptors [16,49,50]. Importantly, we found that following our decellularization protocol, GAGs were retained in our decellularized tissue to a comparable level as the native tissue. This result differs from previous studies in which stronger ionic detergents such as sodium deoxycholate were used to treat rat or porcine small intestines and resulted in significant loss in GAG content [47,51].

A wide range of pepsin-digestion protocols have previously been reported in the literature for tissue sources including adipose tissue, bone, cartilage, colon, small intestine, and heart tissue [18,25–27,29,30,40,52]. Our hydrogels were made from pepsin-digested intestinal ECM using the decellularized mouse tissues. Pepsin digestion requires careful optimization as the peptide solution can be affected by multiple factors including digestion time, pH, enzyme concentration, substrate concentration, temperature, and agitation [25], all factors that can impact hydrogel formation. Indeed, we found that optimization of the ECM concentration was essential to our successful formation of a stable hydrogel, as lower ECM concentrations resulted in unstable hydrogels, while higher ECM concentrations resulted in ineffective digestion. Interestingly, we required higher ECM concentrations for our murine intestinal-derived hydrogels than that previously reported for decellularized porcine tissues [18,25]. This difference in hydrogel stability may be attributable to species-related differences in the ECM composition and/or the decellularization protocols used.

Importantly, the DSI hydrogels generated using our new protocol showed bioactivity by promoting viability and growth of both 3T3 cells and organoids encapsulated in the hydrogels. In comparing intestinal organoids cultured in DSI versus Matrigel®, we also observed similar growth patterns and morphology including an epithelial cell monolayer with budding crypts surrounding a central lumen. These findings are consistent with Giobbe et al, who also reported that intestinal organoids could be maintained in porcine derived ECM over several passages but had no change in their morphology with passaging [18]. We similarly observed that intestinal organoids passaged from DSI back into fresh DSI grew noticeably slower than organoids passaged into Matrigel®. This observation suggests that our DSI gels may need further optimization for expansion of intestinal stem cells when compared to Matrigel®. These data suggest that the DSI gels did not allow for optimal intestinal stem cell maintenance when compared to Matrigel®. Despite retaining collagen, fibronectin, laminin and GAG in our hydrogels, our DSI gels may be lacking in other stem cell niche ECM components, such as tenascin, elastin, or perlecan that have reported to be essential for intestinal stem cells [53–55]. Future studies should apply unbiased mass spectrometry-based techniques to more fully characterize the matrisome composition within the DSI hydrogels compared to Matrigel® [56–58].

Finally, we observed that our DSI hydrogels were less resistant to higher levels of shear stress compared to Matrigel® gels and required careful handling throughout the culture and processing. This was convenient for cell release, but could be an issue for the sustainability of long-term cultures, especially given that matrix stiffness is important for determining intestinal stem cell fate [13,59]. While hydrogels derived from decellularized tissues have been reported to degrade rapidly [40], the DSI hydrogels and Matrigel® were macroscopically able to withstand the 7- and 14-day culture periods with the cell populations used in the current study. However, we acknowledge that the hydrogel composition and biomechanical properties may change over time in culture, and that this could affect stem cell proliferation and differentiation through varying biochemical cues (e.g., cell adhesion or modulation of signalling pathways) [4] and biophysical cues (e.g., stiffness, geometrical guidance and biodegradability) [9,60,61]. Future refinements of these properties, including the addition of synthetic polymers [10] to provide structural reinforcement, other ECM proteins or co-culture with stromal constituents such as myofibroblasts [62] present an opportunity for future optimization of our platform.

One of the limitations of the DSI hydrogels was their lack of tunability when the ECM was applied alone. Thus, we postulated that a composite hydrogel incorporating digested DSI within an alginate carrier could offer advantages including easy cell release by calcium chelation, while allowing greater tuning over the mechanical properties [34]. Incorporation of ECM peptides within alginate can provide the biological cues needed to support cell attachment and growth [31]. However, when mouse intestinal organoids were encapsulated within our alginate-based hydrogels, no cell growth was observed. In a previous study, alginate hydrogels supported the growth of organoids derived from human iPSCs when co-cultured with mesenchymal cells and growth factors such as EGF, R-Spondin2, and Noggin-Fc [31]. However, the number of spheroids that gave rise to organoids was lower in all alginate concentrations used when compared to Matrigel®. Refining the matrix stiffness [63] remains an important limitation of our current DSI platforms. To improve organoid viability and growth, future studies could include varying the alginate concentration, exploring other polymers such as fibrin [64,65], gelatin [66,67] or hyaluronic acid [68,69], as well as the incorporation of growth factors or stromal cells [70,71]. The development of DSI platforms that enable the generation of well-differentiated and consistent intestinal organoid cultures will permit us to explore intestinal diseases and intestinal regeneration. Notably, hydrogel-based systems for organoid culture have been successfully used to create *in vitro* models of intestinal diseases, including inflammatory bowel disease and colorectal cancer [72–74]. In addition, hydrogels have been used to support organoid transplantation to promote intestinal regeneration [3,9].

In summary, although Matrigel® has been used to maintain organoid cultures from several species, there is a clear need to develop new bioscaffold matrices to support the growth of non-tumor organoids. Thus, the DSI hydrogels developed in this study provide a new bioscaffold for the culture and study of cell lines and primary 3D organoids. Our new protocol demonstrates the feasibility of deriving a bioscaffold from the native small intestine of mice and provides a method on which we can build upon to enhance the long-term growth and maintenance of organoids. Future studies will need to focus on altering the DSI gel composition (e.g., adding specific bioactive components or polymers to enable tuning of the biomechanical properties) or organoid culture conditions (e.g., adding intestinal myofibroblasts) in order to further maintain intestinal stem cell cultures long-term.

## Supporting information

S1 Fig H&E staining confirms effective removal of cellular content following decellularization, and rheological testing shows more consistent results in the DSI hydrogel group. A) Representative H&E staining of native and DSI showing effective removal of cellular components while retaining ECM components following decellularization. Black arrows indicate specific intestinal regions including muscularis mucosa, intestinal crypts, and lamina propria (n = 3 cross-sections/decellularization batch, N = 3 independent decellularization batches). Scale bar = 100 μm. B) Rheological testing depicting the storage (G’) and loss (G”) moduli of all DSI hydrogel (a) and Matrigel® (b) samples tested, showing more consistent results in the DSI hydrogels tested (n = 5–6).
(TIF)

S2 Fig Immunofluorescence staining confirmed the retention of ECM components following decellularization. Representative microscopy images of immunofluorescence staining for collagen I, collagen IV, fibronectin, and laminin in native mouse small intestine and following decellularization, demonstrating retention of these markers. All samples were counterstained with DAPI (blue) for cell nuclei. (n = 3 cross-sections/decellularization batch, N = 3 independent decellularization batches). Scale bar = 200 μm. Abbreviations: COL I = collagen type I, COL IV = collagen type IV.
(TIF)

S1 Table Real-Time PCR Primers. (DOCX)
