# Supplementary figures and images for: Development of a tissue-specific bioscaffold for intestinal stem cell culture
Source: PLoS One. 2025 Aug 6;20(8):e0328898. doi: 10.1371/journal.pone.0328898 (PMC12327626; doi:10.1371/journal.pone.0328898)

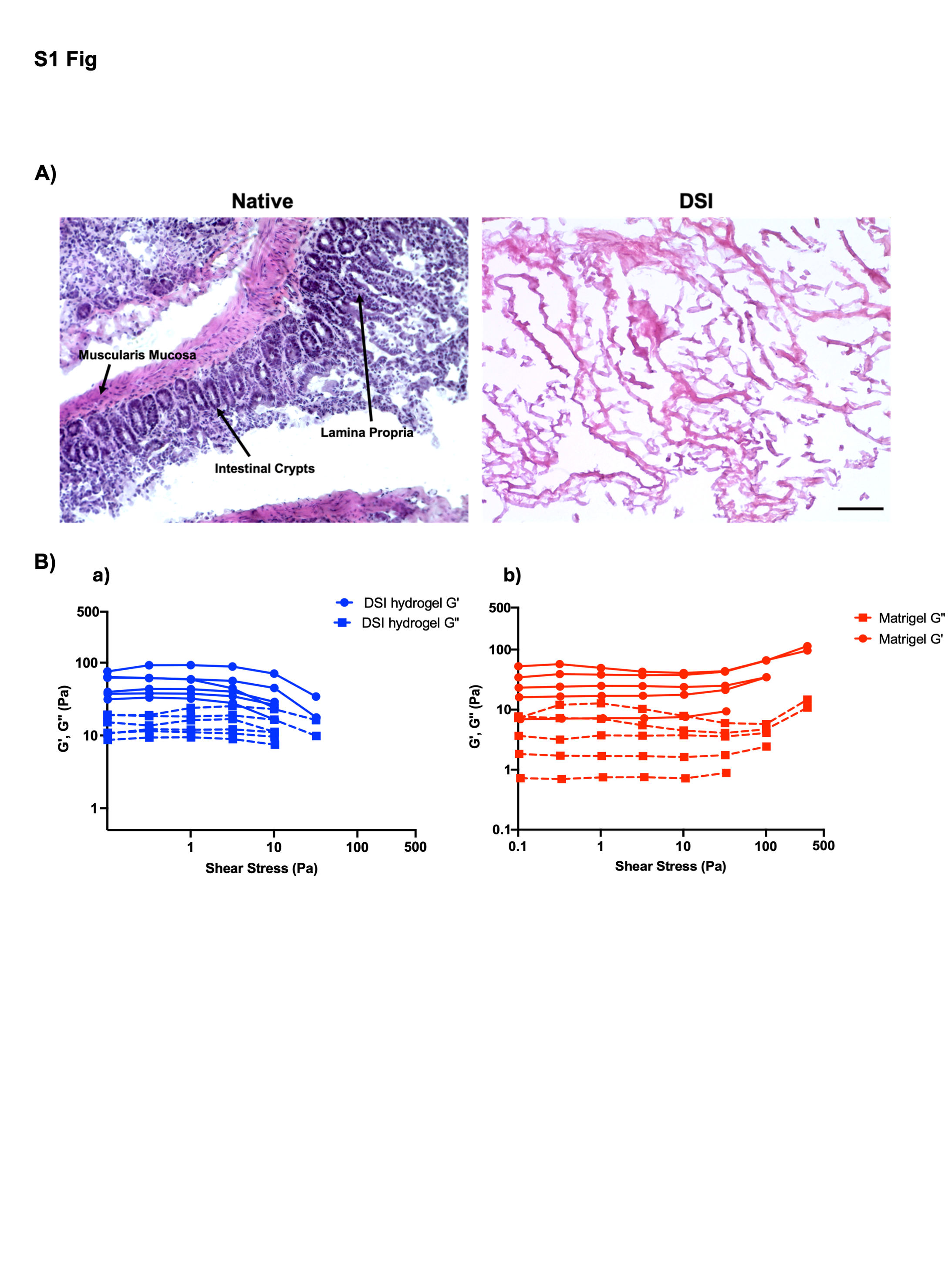

Supplement: S1 Fig — A) Representative H&E staining of native and DSI showing effective removal of cellular components while retaining ECM components following decellularization. Black arrows indicate specific intestinal regions including muscularis mucosa, intestinal crypts, and lamina propria (n = 3 cross-sections/decellularization batch, N = 3 independent decellularization batches). Scale bar = 100 μm. B) Rheological testing depicting the storage (G’) and loss (G”) moduli of all DSI hydrogel (a) and Matrigel® (b) samples tested, showing more consistent results in the DSI hydrogels tested (n = 5–6). (TIF) [file pone.0328898.s001.tif]

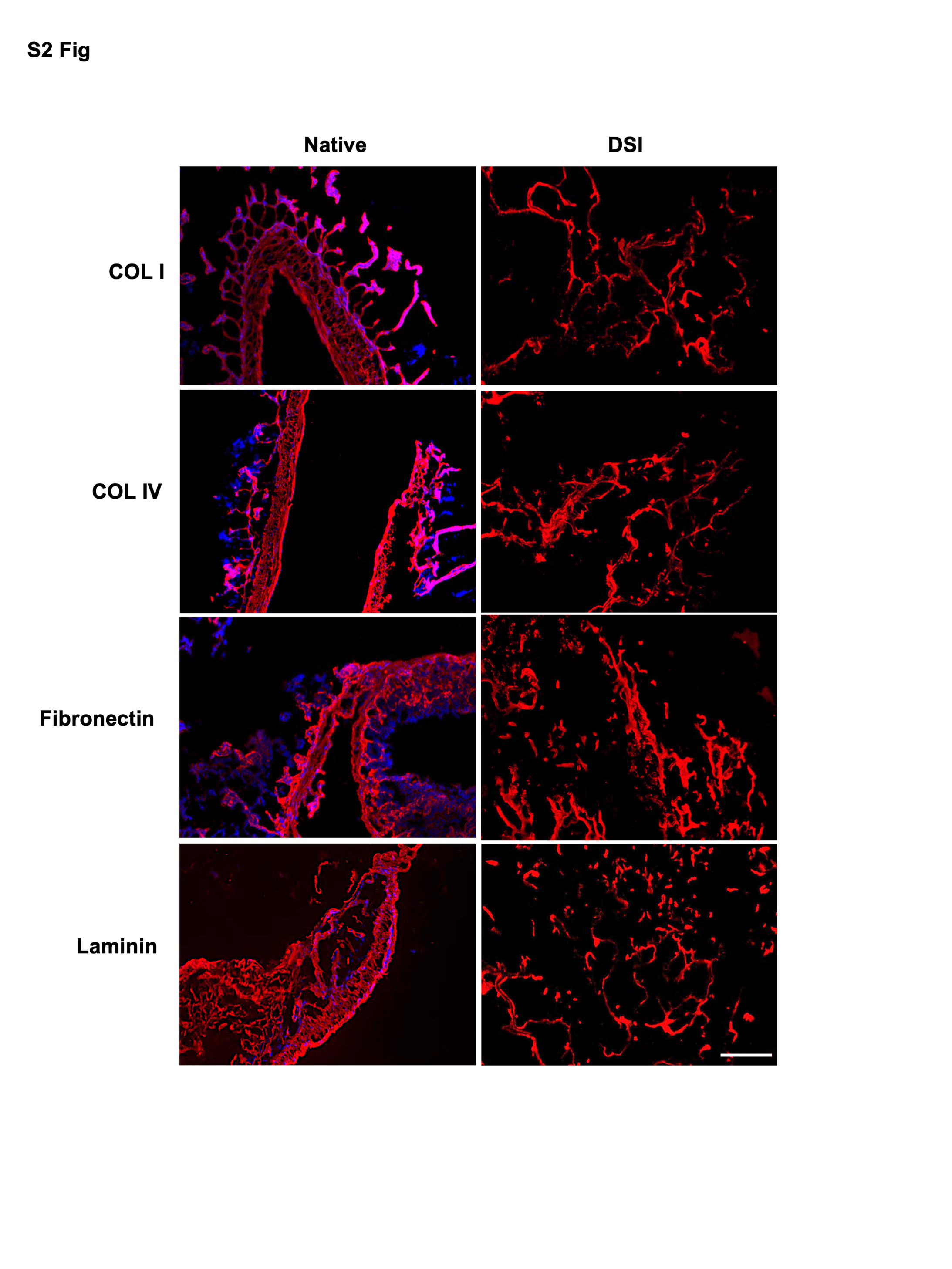

Supplement: S2 Fig — Representative microscopy images of immunofluorescence staining for collagen I, collagen IV, fibronectin, and laminin in native mouse small intestine and following decellularization, demonstrating retention of these markers. All samples were counterstained with DAPI (blue) for cell nuclei. (n = 3 cross-sections/decellularization batch, N = 3 independent decellularization batches). Scale bar = 200 μm. Abbreviations: COL I = collagen type I, COL IV = collagen type IV. (TIF) [file pone.0328898.s002.tif]
