# Supplementary material for: Development of a tissue-specific bioscaffold for intestinal stem cell culture
Source: PLoS One. 2025 Aug 6;20(8):e0328898. doi: 10.1371/journal.pone.0328898 (PMC12327626; doi:10.1371/journal.pone.0328898)
Supplement: S1 Table — (DOCX) [file pone.0328898.s003.docx]

| Gene | Forward | Reverse |
| --- | --- | --- |
| *Gapdh* | GACATCAAGAAGGTGGTGAAGCAG | ATACCAGGAAATGAGCTTGACAAA |
| *Lgr5* | GACGCTGGGTTATTTCAAGTTCAA | CAGCCAGCTACCAAATAGGTGCTC |
| *Dclk1* | AGCACTGCAGCAGGAGTTTCTG | AGTCCTCCGATTCCGAGTTCAA |
| *Muc2* | GCTGACGAGTGGTTGGTGAATG | GATGAGGTGGCAGACAGGAGAC |
| *Lyz1* | TACAACCGTGGAGACCGAAGCA | TGGCTGCAGTGATGTCATCCTG |
| *Chga* | AGAACCAGAGCCCTGATGCCAA | CTCTGTGGTTGCCTCAAAGCCA |
